# Conclusiveness, readability and textual characteristics of plain language summaries from medical and non-medical organizations: a cross-sectional study

**DOI:** 10.1038/s41598-024-56727-6

**Published:** 2024-03-12

**Authors:** Nensi Bralić, Antonija Mijatović, Ana Marušić, Ivan Buljan

**Affiliations:** 1 Department of Research in Biomedicine and Health, University of Split School of Medicine, Šoltanska 2A, 21000 Split, Croatia; 2 Department of Psychology, Faculty of Humanities and Social Sciences, University of Split, Split, Croatia

**Keywords:** Patient education, Public health

## Abstract

This cross-sectional study compared plain language summaries (PLSs) from medical and non-medical organizations regarding conclusiveness, readability and textual characteristics. All Cochrane (medical PLSs, n = 8638) and Campbell Collaboration and International Initiative for Impact Evaluation (non-medical PLSs, n = 163) PLSs of latest versions of systematic reviews published until 10 November 2022 were analysed. PLSs were classified into three conclusiveness categories (conclusive, inconclusive and unclear) using a machine learning tool for medical PLSs and by two experts for non-medical PLSs. A higher proportion of non-medical PLSs were conclusive (17.79% vs 8.40%, P < 0.0001), they had higher readability (median number of years of education needed to read the text with ease 15.23 (interquartile range (IQR) 14.35 to 15.96) vs 15.51 (IQR 14.31 to 16.77), P = 0.010), used more words (median 603 (IQR 539.50 to 658.50) vs 345 (IQR 202 to 476), P < 0.001). Language analysis showed that medical PLSs scored higher for disgust and fear, and non-medical PLSs scored higher for positive emotions. The reason for the observed differences between medical and non-medical fields may be attributed to the differences in publication methodologies or disciplinary differences. This approach to analysing PLSs is crucial for enhancing the overall quality of PLSs and knowledge translation to the general public.

## Introduction

Scientific papers, especially in the medical field, are challenging to read, and their readability has decreased over time^1^. To decide on the best medical treatment option, both medical professionals and patients should be able to understand the health information presented in scientific studies. However, while medical professionals receive training in research methodology and can understand complex medical terms, most patients lack that ability, impeding their involvement in shared decision-making. Even though it is imperative that people without medical education have easy access to health information^2^, several studies have shown that health information is often displayed below the patient’s readability level^3–7^. The American Medical Association recommends that health-related information for patients should be written at or below a 6th grade reading level^8^, and it has been shown that better-written scientific texts result in higher readability and greater comprehension^9^. Kurtzman and Greene^10^ have shown that low-complexity formats can improve patients’ decision-making, which can be achieved using non-technical language and evaluative elements, presenting numerical information in a way that follows the number line (i.e. greater number for more desirable outcomes) and placing them in context.

The volume of evidence in the medical field is increasing exponentially on a daily basis, making it challenging to keep up with the newest discoveries, both by medical professionals and patients^11^. One way to tackle this problem is the development of systematic reviews. Systematic reviews comprehensively analyse the existing evidence for a particular topic, reduce bias, provide strength of evidence assessment, and identify research gaps^12^.

Furthermore, health-related issues have both medical and societal implications, and while they can affect an individual’s health and well-being, they can also impact society as a whole. Health-related issues concerning society include the economic strain on the healthcare system, effectiveness of legal policies, inequalities in access to healthcare, productivity of the healthcare workers and many more topics which are not direct health questions and, therefore, may not be covered by a Cochrane systematic review. However, other organizations, like Campbell Collaboration and International Initiative for Impact Evaluation (3ie), develop systematic reviews and plain language summaries in the social science area^13–15^.

Organizations dedicated to producing high-quality systematic reviews, such as Cochrane, Campbell Collaboration and International Initiative for Impact Evaluation (3ie), work intensively on presenting their results comprehensively to the general public^16–20^. One of the formats used for presenting research study results is the plain language summary (PLS), a summary of the review’s findings written using non-scientific language aimed at the lay audience^21^. Both Cochrane and Campbell Collaborations provide guidance for authors writing a PLS for their systematic review, with the target audience being people without any knowledge of systematic reviews or their topics^20,22^.

Although the readability of Cochrane PLSs is better than that of scientific abstracts written for medical experts^23^, there is room for improvement as the readability levels are still high above the recommended 6th grade readability level^24^. While there are studies assessing the readability of Cochrane systematic review PLSs, there is a lack of such evidence for PLSs published by organizations publishing primarily systematic reviews on social science topics. Stricker et al.^25^ evaluated the readability of PLSs published in two psychology journals, showing that around 17 years of education is needed to understand them. However, they did not specifically include PLSs for systematic reviews.

PLSs serve as a link between the complexities of specialized research studies and the lay audience, making them a critical component of knowledge translation. Conclusiveness of PLSs is critical in this regard, as conclusive systematic reviews give straightforward answers about the effectiveness of the therapy^26^. The potential of PLSs to deliver decisive information not only allows a larger audience to connect with scientific material but also impacts decision-making processes at numerous levels^27^. Several studies assessed the conclusiveness of Cochrane systematic reviews^28–31^ and their PLSs^32^ and found that many reviews and PLSs were inconclusive or had unclear conclusions. However, these studies used a smaller sample size, focused on a single medical field and the coding process was done step-by-step by reviewers, not by utilising technology.

Other important aspects of textual information are its language characteristics, such as emotion and attitude expressed by the author, sentiment of the text and different aspects of the linguistic style, which were shown to impact how individuals perceive its contents^33^. It has been shown that readers’ subjective experiences when processing information determine whether they perceive it as accurate, enjoy it, or have confidence in it^34^. Additionally, language characteristics of the text can induce different emotions and influence how individuals respond and engage with given information^35^, and detecting various language characteristics related to clarity and comprehension of a text can serve as a foundation for evidence-based recommendations for increasing the readability and accessibility of scientific communication materials, which can, in turn, advance the overall idea of encouraging public engagement in scientific research and informed decision-making.

Perković Paloš et al.^36^ compared linguistic and semantic characteristics of articles from social sciences and medicine, showing differences in the word count, clout and tone between the disciplines. However, to our knowledge, no studies have assessed the conclusiveness, readability and language characteristics of PLSs published by non-medical organizations (non-medical PLSs) or compared them to PLSs published by medical organizations (medical PLSs). Our study aimed to explore differences in those outcomes among those PLSs, thus addressing the gap in the literature. By comparing these PLSs, we aimed to gain insight into how scientific information is delivered to different individuals, how this affects the understanding and engagement with the information, as well as the decision-making process. Furthermore, recognizing the linguistic distinctions between PLSs published by medical and non-medical organizations can have far-reaching implications for health communication and knowledge translation.

## Results

The four included organizations published a total of 9476 systematic reviews, of which 9209 were from medical (8928 by Cochrane and 281 by Norwegian Institute of Public Health (NIPH)) and 267 from non-medical organizations (220 by Campbell Collaboration and 47 by 3ie).

Out of 8928 latest published versions of Cochrane systematic reviews, 425 were withdrawn, and 29 had no PLS, resulting in a total of 8474 Cochrane PLSs included in the analysis. NIPH published 281 systematic reviews, of which 32 were in English, and only one had a PLS, which was excluded from the analysis due to sample size disparity and possible sample size bias. Campbell Collaboration published 220 systematic reviews, and 68 had no PLS, resulting in 152 PLSs included in the analysis. 3ie published 47 systematic reviews, and 11 had PLSs, which were included in the analysis.

**Figure 1:**
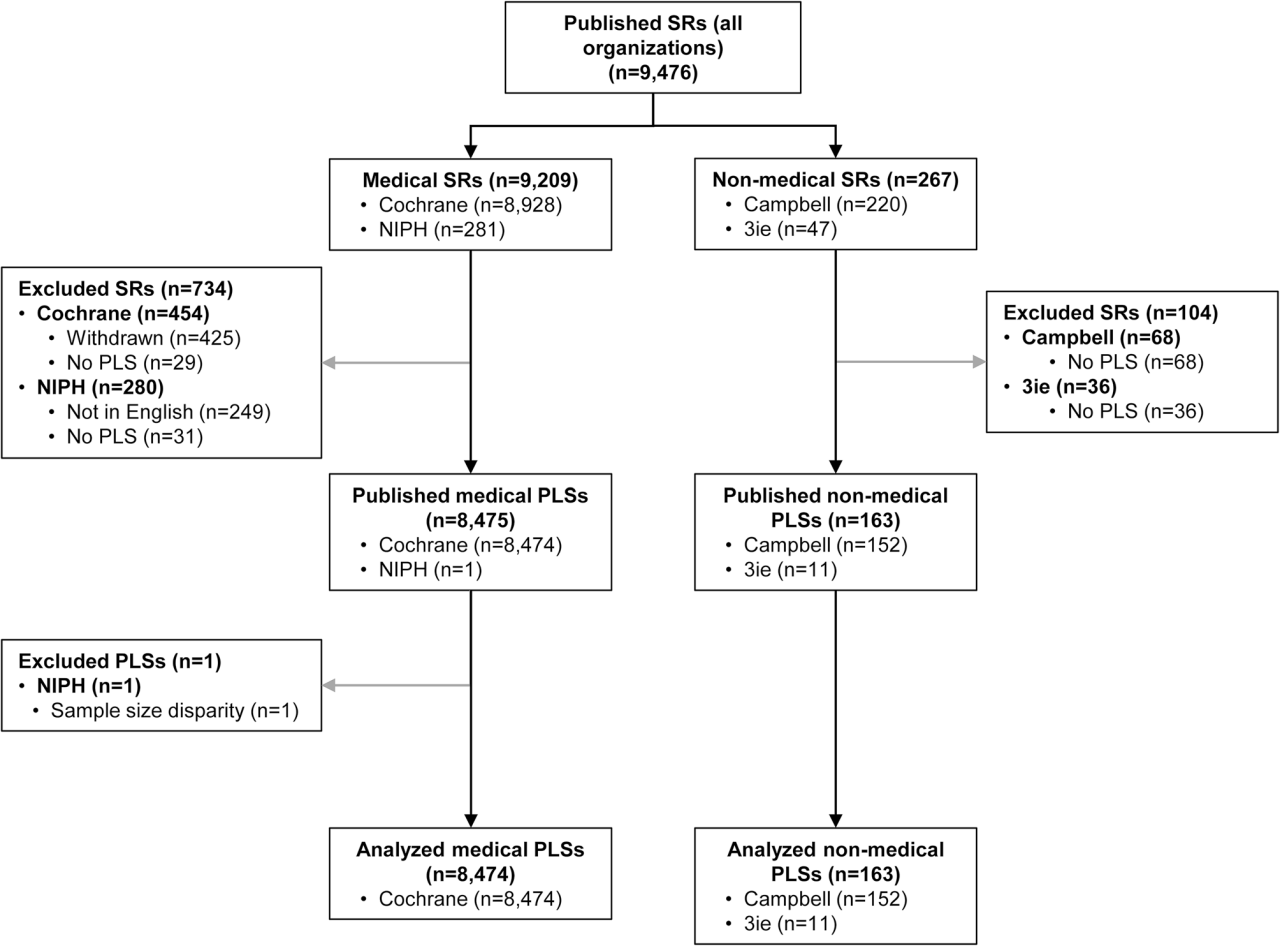
Selection of plain language summaries. SR: systematic review; NIPH: Norwegian Institute of Public Health; 3ie: International Initiative for Impact Evaluation; PLS: plain language summary.

In the end, 8637 PLSs were included in the analysis (8474 from medical and 163 from non-medical organizations) (Fig. 1).

### Conclusiveness

Medical PLSs were largely unclear (62.1%), followed by inconclusive (29.6%) and conclusive (8.4%) conclusions. Non-medical PLSs, on the other hand, were mostly inconclusive (56.4%), followed by unclear (25.8%) and conclusive (17.8%) conclusions.

### Readability

Non-medical PLSs had significantly lower readability levels (i.e. high SMOG scores) compared to medical PLSs (P = 0.010). The median SMOG index score for medical PLSs was 15.51 (95% CI 15.47 to 15.58), whereas the median score for non-medical PLSs was 15.22 (95% CI 14.94 to 15.50). SMOG index score for each conclusiveness category is presented in Supplementary Table S2.

### Language characteristics

**Figure 2:**
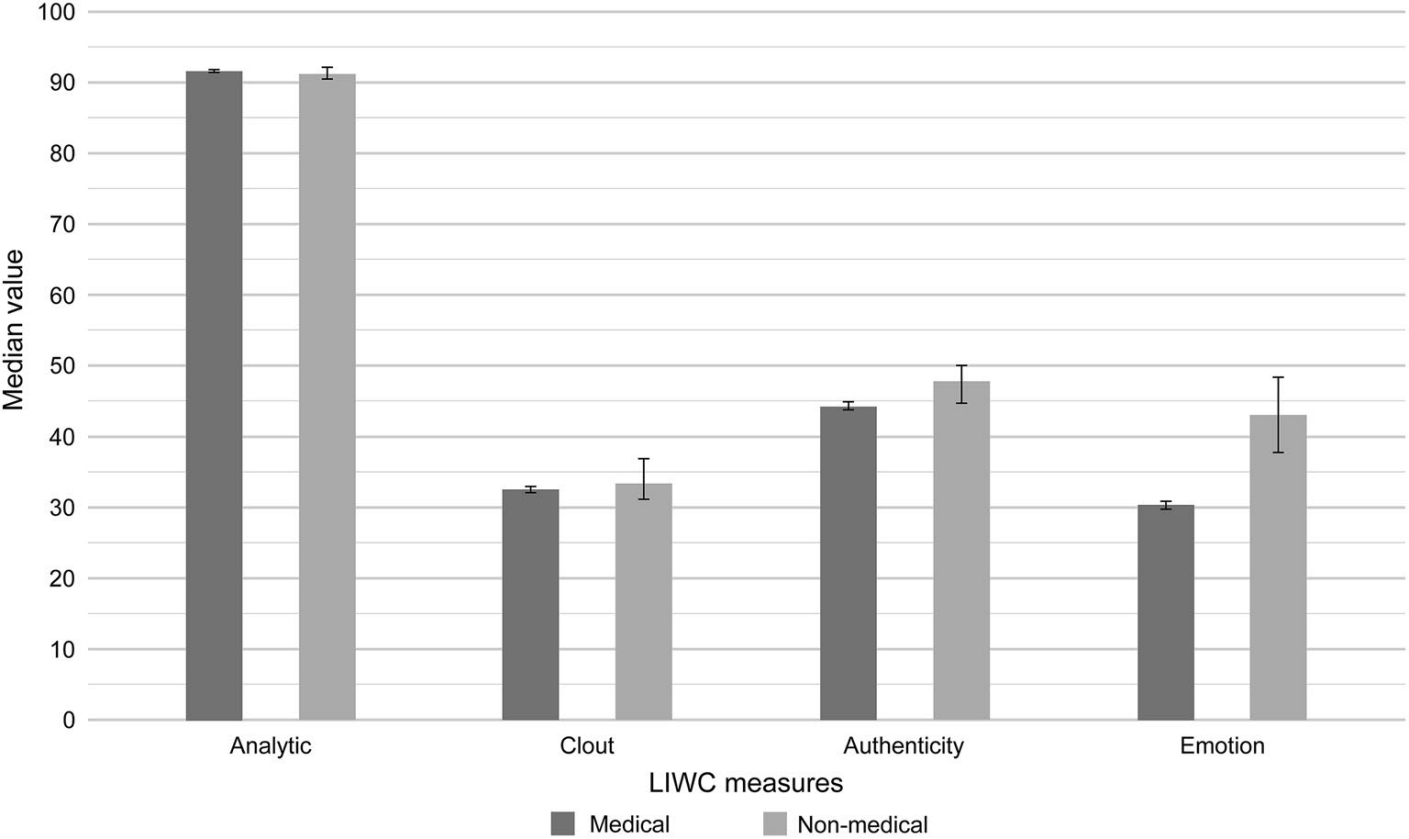
Proportion of specific linguistic characteristics within the PLSs by research domain. Error bars indicate 95% confidence intervals.

Non-medical PLSs were significantly longer than medical PLSs. Additionally, non-medical PLSs scored higher for clout (P = 0.041) and emotional tone (P < 0.001) (Supplementary Tables S5 and S7). There was no difference in the scores for analytical tone between the groups, but medical PLSs with unclear conclusiveness scored lower than other medical PLSs (Supplementary Table S4) (Fig. 2). Conclusive non-medical PLSs had a significantly higher score for ‘clout’ (P = 0.041) and ‘authenticity’ (P = 0.010) than conclusive medical PLSs. Also, inconclusive medical PLSs scored higher for clout, and conclusive medical PLSs scored higher for authenticity than other medical PLSs (Supplementary Tables S5 and S6).

### Linguistic analysis

**Figure 3:**
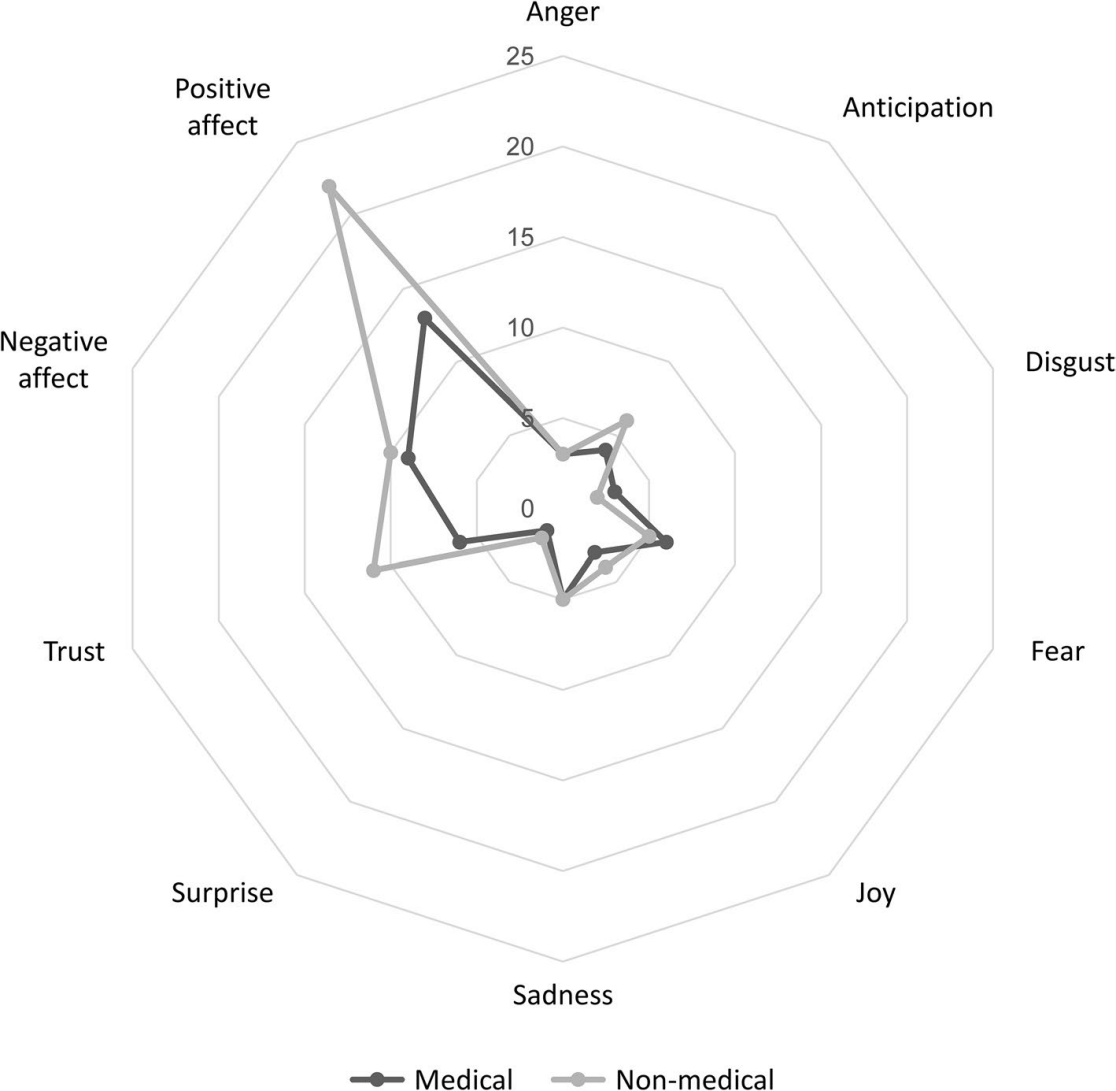
Sentiment analysis of the PLSs from medical and non-medical research fields.

Non-medical PLSs scored higher for both positive and negative sentiments compared to medical PLSs. Also, non-medical PLSs had a higher presence of emotions such as anger, anticipation, joy, surprise, and trust, while medical PLSs scored higher for disgust and fear (Fig. 3).

The logistic model showed multiple characteristics predicting whether a PLS is medical or non-medical (Table 1). PLSs with a lower SMOG index (OR = 0.808; 95% CI 0.721 to 0.905; P < 0.001) were more likely to be non-medical, as well as PLSs with higher word count (OR = 1.005; 95% CI 1.003 to 1.006; P < 0.001), although the difference was practically not relevant. Additionally, higher scores for analytic tone and emotions such as anger, trust and positive sentiment were associated with non-medical PLSs, whereas disgust, fear and joy predicted medical PLSs. Nine factors were included in the logistic regression, describing 36.9% of the variance.

**Table 1:** Logistic regression model of characteristics predicting the criteria (medical field vs non-medical field).

Variables	OR (95% CI)	P-value
SMOG index	0.808 (0.721 to 0.905)	< 0.001
Word count	1.005 (1.003 to 1.006)	< 0.001
Analytic tone	1.074 (1.040 to 1.109)	< 0.001
Anger	1.673 (1.477 to 1.895)	< 0.001
Disgust	0.698 (0.609 to 0.800)	< 0.001
Fear	0.617 (0.562 to 0.677)	< 0.001
Joy	0.873 (0.789 to 0.966)	0.009
Trust	1.204 (1.120 to 1.293)	< 0.001
Positive sentiment	1.102 (1.047 to 1.161)	< 0.001

Type level ‘Non-medical’ coded as class 1, and ‘Medical’ as class 0.

*OR* odds ratio, *CI* confidence interval.

McFadden R^2^—0.369.

## Discussion

Our cross-sectional, methodological study showed significant differences between medical and non-medical PLSs regarding conclusiveness, readability and textual characteristics. Non-medical PLSs were more conclusive, and although both groups had readability levels significantly above the recommended 6th grade level, non-medical PLSs had greater readability than medical PLSs but were written with significantly more words.

The difference in conclusiveness might originate from the different objectives of the two research fields, as the medical field often deals with life-and-death decisions and human body variability regarding genetics, treatment response and disease progression. For that reason, researchers from the medical field might be more careful and reserved when making conclusions about a particular treatment option. Additionally, lower conclusiveness in medical PLSs should not always be considered a weakness, as it might simply be an accurate representation of the current state of medical evidence. These findings align with other studies that found low conclusiveness in Cochrane Reviews^28–31^ as well as their PLSs^32^. The overall lack of studies assessing the conclusiveness of non-medical PLSs was addressed in our study and, together with the application of machine learning tool, presents a strength of the study.

The observed difference in the word count between the two categories of PLSs may indicate different communication strategies, with non-medical PLSs prioritising comprehensive explanations and using words to ensure clarity and completeness. In contrast, medical PLSs might focus on conciseness without overwhelming the reader. Another possible explanation could be that non-medical PLSs were more heterogeneous or measured more outcomes compared to medical PLSs.

There was also a difference between Cochrane and Campbell guidelines regarding the recommended word limits for their PLSs^20,22^. Cochrane advises that the PLSs be written within 400 to 850 words, while Campbell suggests a target length of 600 to 750 words. A qualitative analysis is needed to determine the reasons for the word count discrepancy, which should be considered in future studies.

Regarding linguistic characteristics, non-medical PLSs had higher scores for LIWC dimensions clout, emotional tone, anger, anticipation, joy, surprise and trust. In comparison, medical PLSs had higher scores for fear and disgust. These results indicate that non-medical PLSs contain more words and phrases that reflect confidence and social influence. Still, there was also a higher presence of emotional and affective words and phrases indicating positive or negative emotions. Further research is needed to determine the most engaging tone and simplification of the PLSs for the readers. The potential for development possibly lies in the use of language models such as ChatGPT (https://chat.openai.com/) or BERT (10.48550/arXiv.1810.04805), for which it is expected to be more incorporated into everyday life in the future.

Additionally, non-medical PLSs contained more words and phrases that convey a sense of having a positive outlook towards the future, as well as a sense of astonishment and faith in someone or something. Medical PLSs, on the other hand, contained more words associated with anxiety and expressions of fear, as well as words that suggest strong aversion and distaste. This could be due to the fact that medical PLSs contain more words associated with the words pain and disease, which could contribute to the difference in the level of negative emotions. Still, the presence of those words cannot explain the greater prevalence of positive words. The recommendation for future studies is to employ a large-scale qualitative analysis approach to make a firmer conclusion.

Although non-medical PLSs had statistically higher readability than medical PLSs, both were still written significantly above the recommended 6th grade reading level^8^. The aim of PLSs is to present advanced constructs to the end-users who do not necessarily have the knowledge to comprehend the complete research article^37^, and maintaining the readability level at or below the 6th grade standard is critical to avoid excluding a large proportion of the intended audience. These findings align with studies by Banić et al.^32^ and Karačić et al.^24^, which also found that around 15 years of education was needed to comprehend a Cochrane PLS. Moreover, when simplifying language in PLSs, writers should be careful not to change the conclusions of the PLSs, so further study is needed on whether downgrading the reading levels creates changes in the quality of the message in a PLS.

Our results should be interpreted in view of several limitations. The first relates to the difference in the number of PLSs in each group. Cochrane is the most prominent organization publishing PLSs for systematic reviews in the field of medicine, and PLSs are mandatory for all systematic reviews in the Cochrane Database of Systematic Reviews^38^. Other scientific fields have not yet systematically adopted this practice. Since the medical field contributes the majority of systematic reviews nowadays, this could explain the significant difference in the number of published PLSs between the two groups.

Another limitation is the fact that we originally used the machine learning tool for the classification of conclusiveness for both medical and non-medical PLSs. However, after double-checking the output, we found the tool to be imprecise for non-medical PLSs. This could be because the machine learning tool was trained on medical texts, making it imprecise in determining the conclusiveness of the non-medical PLSs. Therefore, to mitigate the introduction of potential biases and ensure accuracy, non-medical PLSs were classified by hand by one author and verified by the second author. Also, we classified PLSs into medical and non-medical categories solely based on the organization that published them. This might not be entirely correct as Campbell Collaboration and 3ie occasionally publish systematic reviews dealing with medical topics. Topics most covered by non-medical organizations include social and behavioural interventions, societal issues and policies, workplace safety and efficiency, educational interventions, well-being and social services, and criminal justice and enforcement.

Our study focused only on the characteristics of the PLSs. Future studies could explore how the readability of PLS could impact health literacy outcomes for different population members. Also, future studies could assess how the quality and readability of PLSs affect an individual’s healthcare decision and include the perspectives of the target audiences to enhance the accessibility of research findings.

In conclusion, there are differences between medical and non-medical PLSs regarding conclusiveness, word count, readability and textual characteristics, and the reason for these differences is still unknown. However, they may be attributed to the differences in publication methodologies or disciplinary differences. Both medical and non-medical PLSs are still written below the recommended readability level, which could contribute to limited comprehension, the spread of misinformation and the exclusion of a part of the target audience. It is a question of whether the recommended level of reading ease is possible without changing the conclusions. Future studies could explore the role of large language models in writing messages for the public to save time and resources and improve their readability. Overall, our study might have important implications for PLS readers by assisting in increasing comprehension of and engagement with the scientific information, as well as improving their decision-making abilities.

## Methods

### Aim

This study aimed to compare the conclusiveness, readability, and textual characteristics of PLSs between medical and non-medical (social) organizations.

### Study design, settings, and eligible summaries

In our cross-sectional, methodological, research-on-research study, we included all medical PLSs of the latest versions of systematic reviews in the English language published by the Cochrane and the Norwegian Institute of Public Health (NIPH), and non-medical PLSs from the Campbell Collaboration and the International Initiative for Impact Evaluation (3ie) from inception until 10 November 2022. Systematic reviews that used Cochrane methodology but were not Cochrane systematic reviews published in the Cochrane Library were not included. The protocol for this study was registered on the Open Science Framework prior to study commencement (https://osf.io/2kvs3). We planned to include PLSs from two more organizations (Joanna Briggs Institute and Evidence for Policy and Practice Information and Co-ordinating Centre). However, during the data collection phase, we discovered that those organizations have not yet published PLSs for their systematic reviews.

### Data collection

An electronic mail was sent to the representatives of all six included organizations asking for all PLSs of their published systematic reviews. We received two replies. A representative of the Joanna Briggs Institute informed us that their organization does not publish PLSs, and a representative from Cochrane instructed us to submit our request via the data request form on their web page. We used the web-scraping method through *rvest*^39^ and *tidyverse*^40^ packages in R software version 4.2.1. (R Core Team, 2020)^41^ to retrieve titles, links, and publication dates of systematic reviews from the Campbell Collaboration (https://www.campbellcollaboration.org/), NIPH (https://www.fhi.no/) and 3ie (https://www.3ieimpact.org/) web pages. The PLSs from the Campbell Collaboration were retrieved using web-scraping, while those from the NIPH and 3ie were retrieved manually. Cochrane PLSs were retrieved using the “Export selected citation(s)” option in Cochrane Library, and titles, links, publication dates and PLSs were extracted from the citations using R package *stringr*^42^*.* We followed the STROBE guidelines for reporting (Supplementary Table S19).

### Outcomes

#### Conclusiveness

PLSs were classified as conclusive, inconclusive or unclear using the fine-tuned large language model based on SciBERT, a pre-trained language model for scientific text^43^, which we validated and trained on medical PLSs^32^. A large language model was used to enhance efficiency and ensure consistency by minimizing human error. After double-checking model inputs for non-medical PLSs, we found it inaccurate in classifying non-medical PLSs. Therefore, for the non-medical PLSs, one author rated the conclusiveness, while the other author verified this rating according to the three conclusiveness categories:

1. Conclusive
  Positive—There is moderate or high-quality evidence indicating the effectiveness or safety.
  Negative—There is moderate or high-quality evidence indicating that the intervention is ineffective or harmful.
  Equal—The interventions analysed were equally effective and safe.
2. Inconclusive
  Positive inconclusive—There is evidence suggesting effectiveness or safety, but it is of low quality or inconclusive, and the authors suggest that more research is needed.
  Negative inconclusive—There is evidence of ineffectiveness or harm (evidence demonstrating that there was no effect or that the intervention was not safe) or authors urged against the intervention or comparison, or it is not recommended; however, the evidence is of low quality or inconclusive, or authors state that more research is needed.
  Equal inconclusive—The interventions appear to be similarly effective and safe, but the evidence is of lower quality or inconclusive, and the authors suggest that more research is needed.
3. Unclear
  No evidence—There is no evidence as the search did not retrieve any randomized controlled trials, i.e. empty reviews.
  No opinion—The authors did not offer any opinion or judgment.
  Unclear—The authors did not give a clear conclusion.

### Readability

Readability was assessed in R, using the Simple Measure of Gobbledygook (SMOG) index readability score^44^. The SMOG index measures how many years of education are needed for an average person to comprehend the text. The American Medical Association recommends that written materials with health information for patients should be written at or below the 6th grade reading level^8^.

### Language characteristics

Linguistic Inquiry and Word Count (LIWC) text analysis software was used to analyze the textual characteristics of PLSs. Aside from the word count for each PLS, LIWC calculates the percentage of words in the PLSs that match different dictionary categories (Analytical tone, Clout, Authenticity and Emotional tone). The analytical tone variable indicates an objective writing style, with higher scores implying the text was written more formally, rationally and hierarchically. The clout variable is associated with confidence and assertiveness, with lower scores indicating a more hesitant text tone. A higher authenticity score suggests the use of more first-person pronouns, singular forms, present tense verbs and relativity terms. Additionally, when the text projects more positive emotions, the emotional tone score rises. Sentiment analysis was done using LIWC and the *syuzhet* package in R^45^. The *get_NRC_sentiment* function in the *syuzhet* package acts as a dictionary-based sentiment analysis tool, providing scores on eight different emotion categories associated with the text as well as positive and negative valence. The function counts words found in the NRC’s lexicon for each category. Emotional categories included anger, anticipation, disgust, fear, joy, sadness, surprise and trust.

### Statistical analysis

We presented the data on conclusiveness as frequencies and percentages. Numerical data were checked for normality using the Shapiro–Wilk test and presented as medians with 95% confidence intervals. SMOG index was presented as a median with 95% CI. LIWC variables were described as scores from 0 to 100, which indicates the percentage of the words in a given text related to a specific LIWC category.

Chi-squared test was used to compare the scores on conclusiveness within groups, as well as within conclusiveness categories. Mann–Whitney test was used to test the differences in the scores for the SMOG index, LIWC variables and sentiment analysis variables between the groups. Kruskal-Walls test was used to test the differences in the scores between the three conclusiveness categories.

A logistic regression model was created using a stepwise method, including the most significant variables as predictors of the criteria (medical field vs non-medical field). The regression results were presented with odds ratios (ORs), 95% confidence intervals and McFadden R^2^. All analyses were performed using MedCalc software, version 20.027 (MedCalc Software, Ostend, Belgium).

### Preregistration

This study has been registered on the Open Science Framework prior to study commencement (https://osf.io/2kvs3).

### Supplementary Information

Supplementary Information.

## Data Availability

Raw data for this study is available on the OSF platform (https://osf.io/ugfvd).
