# Supplementary material for: Conclusiveness, readability and textual characteristics of plain language summaries from medical and non-medical organizations: a cross-sectional study
Source: Sci Rep. 2024 Mar 12;14:6016. doi: 10.1038/s41598-024-56727-6 (PMC10933350; doi:10.1038/s41598-024-56727-6)
Supplement: Supplementary file 1 — Supplementary Information. [file 41598_2024_56727_MOESM1_ESM.pdf]

Title: Conclusiveness, readability and textual characteristics of plain language summaries from medical and non-medical organisations: a cross-sectional study

Authors: Nensi Bralić, DMD[a]*, Antonija Mijatović, PhD[a], Ana Marušić, MD, PhD[a], Ivan Buljan, PhD[b]

Affiliations:

[a]Department of Research in Biomedicine and Health, University of Split School of Medicine, Split, Croatia, nensi.bralic@mefst.hr, antonija.mijatovic@mefst.hr, ana.marusic@mefst.hr

[b]Department of Psychology, Faculty of Humanities and Social Sciences, University of Split, Split, Croatia, ibuljan@ffst.hr

***Corresponding author:**

Nensi Bralić, DMD

Department of Research in Biomedicine and Health, University of Split School of Medicine, Šoltanska 2, 21000 Split, Croatia

Tel.: +385 91 914 0400

Email: nensi.bralic@mefst.hr

Declaration of interest: none

Supplementary Table S1. Distribution of PLSs between groups based on the conclusiveness categories.

Variables N(%)	Overall sample (n=8,637)	Medical (n=8,474)	Non-medical (n=163)	P*
Conclusive	739 (8.56)	710 (8.38)	29 (17.79)	**<0.001**
Inconclusive	2,597 (30.07)	2,505 (29.56)	92 (56.44)	**<0.001**
No opinion	5,301 (61.38)	5,259 (62.06)	42 (25.77)	**<0.001**
P*	**<0.001**	**<0.001**	**<0.0001**	

Supplementary Table S2. SMOG index depending on the conclusiveness category

Variables (Md. 95% CI)	Overall sample (n=8,637)	Medical (n=8,474)	Non-medical (n=163)	P*
Conclusive	15.76 (15.58 to 15.90) ‡	15.78 (15.58 to 15.90) ‡	15.25 (14.59 to 15.57)	0.058
Inconclusive	15.61 (15.50 to 15.68) ‡	15.63 (15.53 to 15.71) ‡	15.16 (14.70 to 15.49)	**0.005**
No opinion	15.44 (15.38 to 15.49) ‡	15.44 (15.38 to 15.49) ‡	15.35 (14.97 to 15.70)	0.551
P†	**<0.001**	**<0.001**	0.799	

*Mann-Whitney test.
†Kruskal-Wallis test.
‡Statistically significant from other groups

Supplementary Table S3. Word count depending on the conclusiveness category

Variables (Md. 95% CI)	Overall sample (n=8,637)	Medical (n=8,474)	Non-medical (n=163)	P*
Conclusive	164.00 (158.00 to 175.00) ‡	161.00 (153.00 to 168.60) ‡	613.00 (561.96 to 653.11)	**<0.001**
Inconclusive	387.00 (379.00 to 393.00) ‡	380.00 (369.00 to 387.00) ‡	616.00 (580.25 to 629.00)	**<0.001**
No opinion	362.00 (354.00 to 368.00) ‡	360.00 (352.00 to 367.00) ‡	596.50 (561.66 to 631.26)	**<0.001**
P†	**<0.001**	**<0.001**‡	0.817	

*Mann-Whitney test.
†Kruskal-Wallis test.
‡Statistically significant from other groups

Supplementary Table S4. Analytical tone depending on the conclusiveness category

Variables (Md. 95% CI)	Overall sample (n=8,637)	Medical (n=8,474)	Non-medical (n=163)	P*
Conclusive	93.18 (92.41 to 93.63)	93.23 (92.45 to 93.74)	91.73 (89.62 to 93.22)	0.206
Inconclusive	92.43 (92.22 to 92.70)	92.53 (92.24 to 92.75)	91.31 (90.22 to 92.42)	0.244
No opinion	91.22 (91.03 to 91.46) ‡	91.23 (91.03 to 91.46) ‡	91.18 (90.07 to 93.32)	0.236
P†	**<0.001**	**<0.001**	0.798	

*Mann-Whitney test.
†Kruskal-Wallis test.
‡Statistically significant from other groups

Supplementary Table S5. Clout depending on the conclusiveness category

Variables (Md. 95% CI)	Overall sample (n=8,637)	Medical (n=8,474)	Non-medical (n=163)	P*
Conclusive	31.88 (30.11 to 33.95)	31.52 (29.87 to 33.63)	39.11 (31.76 to 46.59)	**0.041**
Inconclusive	34.02 (33.35 to 34.72) ‡	34.03 (33.37 to 34.80) ‡	33.12 (28.66 to 38.53)	0.850
No opinion	32.01 (31.46 to 32.42)	32.00 (31.46 to 32.42)	32.79 (29.69 to 37.17)	0.270
P†	**<0.001**	**<0.001**	0.436	

*Mann-Whitney test.
†Kruskal-Wallis test.
‡Statistically significant from other groups

Supplementary Table S6. Authenticity depending on the conclusiveness category

Variables (Md. 95% CI)	Overall sample (n=8,637)	Medical (n=8,474)	Non-medical (n=163)	P*
Conclusive	37.49 (35.52 to 40.36) ‡	36.85 (35.05 to 39.59) ‡	49.41 (40.72 to 56.33)	**0.010**
Inconclusive	45.08 (43.95 to 45.99)	44.89 (43.81 to 45.96)	46.88 (43.68 to 53.04)	0.316
No opinion	44.91 (44.31 to 45.69)	44.91 (44.30 to 45.67)	46.15 (42.46 to 50.19)	0.521
P†	**<0.001**	**<0.001**	0.876	

*Mann-Whitney test.
†Kruskal-Wallis test.
‡Statistically significant from other groups

Supplementary Table S7. Emotional tone depending on the conclusiveness category

Variables (Md. 95% CI)	Overall sample (n=8,637)	Medical (n=8,474)	Non-medical (n=163)	P*
Conclusive	31.55 (29.40 to 33.16)	31.09 (28.66 to 32.67)	51.62 (38.92 to 64.32)	**0.002**
Inconclusive	29.89 (28.96 to 30.96)	29.67 (28.64 to 30.60)	39.84 (33.85 to 44.28)	**0.008**
No opinion	30.85 (30.05 to 31.61)	30.73 (29.97 to 31.54)	46.32 (36.26 to 57.74)	**0.002**
P†	0.464	0.350	0.118	

*Mann-Whitney test.
†Kruskal-Wallis test.

Supplementary Table S8. Anger depending on the conclusiveness category

Variables (Md. 95% CI)	Overall sample (n=8,637)	Medical (n=8,474)	Non-medical (n=163)	P*
Conclusive	2.00 (2.00 to 2.00) ‡	2.00 (2.00 to 2.00) ‡	3.00 (2.77 to 4.00)	**0.001**
Inconclusive	3.00 (3.00 to 3.00)	3.00 (3.00 to 3.00)	4.00 (3.00 to 4.00)	**0.042**
No opinion	3.00 (3.00 to 3.00)	3.00 (3.00 to 3.00)	3.00 (3.00 to 4.00)	0.127
P†	**<0.001**	**<0.001**	0.764	

*Mann-Whitney test.
†Kruskal-Wallis test.
‡Statistically significant from other groups

Supplementary Table S9. Anticipation depending on the conclusiveness category

Variables (Md. 95% CI)	Overall sample (n=8,637)	Medical (n=8,474)	Non-medical (n=163)	P*
Conclusive	2.00 (2.00 to 3.00) ‡	2.00 (2.00 to 3.00) ‡	6.00 (4.00 to 7.00)	**<0.001**
Inconclusive	4.00 (4.00 to 5.00) ‡	4.00 (4.00 to 5.00) ‡	7.00 (6.00 to 7.00)	**<0.001**
No opinion	4.00 (4.00 to 4.00) ‡	4.00 (4.00 to 4.00) ‡	6.00 (5.00 to 7.00)	**<0.001**
P†	**<0.001**	**<0.001**	0.309	

*Mann-Whitney test.
†Kruskal-Wallis test.
‡Statistically significant from other groups

Supplementary Table S10. Disgust depending on the conclusiveness category

Variables (Md. 95% CI)	Overall sample (n=8,637)	Medical (n=8,474)	Non-medical (n=163)	P*
Conclusive	2.00 (2.00 to 2.00) ‡	2.00 (2.00 to 2.00) ‡	1.00 (0.77 to 3.00)	0.275
Inconclusive	3.00 (3.00 to 3.00)	3.00 (3.00 to 3.00)	2.00 (2.00 to 2.00)	**0.006**
No opinion	3.00 (2.00 to 3.00)	3.00 (3.00 to 3.00)	2.00 (2.00 to 2.00)	**0.003**
P†	**<0.001**	**<0.001**	0.243	

*Mann-Whitney test.
†Kruskal-Wallis test.
‡Statistically significant from other groups

Supplementary Table S11. Fear depending on the conclusiveness category

Variables (Md. 95% CI)	Overall sample (n=8,637)	Medical (n=8,474)	Non-medical (n=163)	P*
Conclusive	4.00 (4.00 to 4.00) ‡	4.00 (4.00 to 4.00) ‡	4.00 (1.77 to 6.23)	0.932
Inconclusive	6.00 (6.00 to 6.00)	6.00 (6.00 to 6.00)	5.00 (4.13 to 6.00)	**0.019**
No opinion	6.00 (6.00 to 6.00)	6.00 (6.00 to 6.00)	5.00 (3.00 to 6.00)	**0.036**
P†	**<0.001**	**<0.001**	0.443	

*Mann-Whitney test.
†Kruskal-Wallis test.
‡Statistically significant from other groups

Supplementary Table S12. Joy depending on the conclusiveness category

Variables (Md. 95% CI)	Overall sample (n=8,637)	Medical (n=8,474)	Non-medical (n=163)	P*
Conclusive	2.00 (2.00 to 2.00) ‡	2.00 (2.00 to 2.00) ‡	3.00 (2.00 to 5.23)	**<0.001**
Inconclusive	3.00 (3.00 to 3.00) ‡	3.00 (3.00 to 3.00)	4.00 (4.00 to 5.00)	**<0.001**
No opinion	3.00 (3.00 to 3.00) ‡	3.00 (3.00 to 3.00)	4.00 (3.00 to 5.00)	**<0.001**
P†	**<0.001**	**<0.001**	0.475	

*Mann-Whitney test.
†Kruskal-Wallis test.
‡Statistically significant from other groups

Supplementary Table S13. Sadness depending on the conclusiveness category

**Variables (Md. 95% CI)**	**Overall sample (n=8,637)**	**Medical (n=8,474)**	**Non-medical (n=163)**	**P***
Conclusive	3.00 (3.00 to 3.00) ‡	3.00 (3.00 to 3.00) ‡	4.00 (3.00 to 5.00)	**0.044**
Inconclusive	5.00 (5.00 to 5.00)	5.00 (5.00 to 5.00)	5.00 (4.00 to 5.88)	0.834
No opinion	5.00 (5.00 to 5.00)	5.00 (5.00 to 5.00)	5.00 (4.00 to 6.00)	0.828
P†	**<0.001**	**<0.001**	0.234	

*Mann-Whitney test.
†Kruskal-Wallis test.
‡Statistically significant from other groups

Supplementary Table S14. Surprise depending on the conclusiveness category

**Variables (Md. 95% CI)**	**Overall sample (n=8,637)**	**Medical (n=8,474)**	**Non-medical (n=163)**	**P***
Conclusive	1.00 (1.00 to 1.00) ‡	1.00 (1.00 to 1.00) ‡	1.00 (0.00 to 2.00)	0.385
Inconclusive	2.00 (2.00 to 2.00)	2.00 (2.00 to 2.00)	2.00 (2.00 to 2.00)	**0.006**
No opinion	2.00 (2.00 to 2.00)	2.00 (2.00 to 2.00)	2.00 (1.00 to 3.00) ‡	0.170
P†	**<0.001**	**<0.001**	**0.010**	

*Mann-Whitney test.
†Kruskal-Wallis test.
‡Statistically significant from other groups

Supplementary Table S15. Trust depending on the conclusiveness category

**Variables (Md. 95% CI)**	**Overall sample (n=8,637)**	**Medical (n=8,474)**	**Non-medical (n=163)**	**P***
Conclusive	3.00 (3.00 to 4.00) ‡	3.00 (3.00 to 4.00) ‡	13.00 (11.00 to 16.00) §	**<0.001**
Inconclusive	6.00 (6.00 to 7.00)	6.00 (6.00 to 6.00)	10.50 (9.13 to 12.00)	**<0.001**
No opinion	6.00 (6.00 to 6.00)	6.00 (6.00 to 6.00)	11.50 (10.00 to 14.00)	**<0.001**
P†	**<0.001**	**<0.001**	0.050	

*Mann-Whitney test.
†Kruskal-Wallis test.
‡Statistically significant from other groups
§Statistically significant from inconclusive group

Supplementary Table S16. Negative attitude depending on the conclusiveness category

**Variables (Md. 95% CI)**	**Overall sample (n=8,637)**	**Medical (n=8,474)**	**Non-medical (n=163)**	**P***
Conclusive	6.00 (5.00 to 6.00) ‡	6.00 (5.00 to 6.00) ‡	7.00 (6.00 to 10.00) §	**0.003**
Inconclusive	9.00 (9.00 to 9.00)	9.00 (9.00 to 9.00)	11.00 (9.13 to 12.00)	**0.001**
No opinion	9.00 (9.00 to 9.00)	9.00 (9.00 to 9.00)	10.00 (8.19 to 11.82)	0.097
P†	**<0.001**	**<0.001**	**0.026**	

*Mann-Whitney test.
†Kruskal-Wallis test.
‡Statistically significant from other groups
§Statistically significant from inconclusive group

Supplementary Table S17. Positive attitude depending on the conclusiveness category

Variables (Md. 95% CI)	Overall sample (n=8,637)	Medical (n=8,474)	Non-medical (n=163)	P*
Conclusive	7.00 (6.00 to 7.00) ‡	6.00 (6.00 to 7.00) ‡	21.00 (19.77 to 27.23)	**<0.001**
Inconclusive	14.00 (13.00 to 14.00) ‡	14.00 (13.00 to 14.00) ‡	22.00 (20.00 to 23.00)	**<0.001**
No opinion	13.00 (13.00 to 13.00) ‡	13.00 (13.00 to 13.00) ‡	23.00 (19.19 to 25.82)	**<0.001**
P†	**<0.001**‡	**<0.001**‡	0.581	

*Mann-Whitney test.
†Kruskal-Wallis test.
‡Statistically significant from other groups

Supplementary Table S18. Scores for both groups for all measured variables

	Medical PLSs (n=8,474) Md (95% CI)	Non-Medical PLSs (n=163) Md (95% CI)	P*
SMOG index	15.51 (15.47 to 15.58)	15.23 (14.94 to 15.50)	0.010
Analytic	91.76 (91.57 to 91.97)	91.22 (90.56 to 92.24)	0.788
Clout	32.62 (32.19 to 33.04)	33.41 (31.25 to 36.95)	0.042
Authentic	44.29 (43.87 to 44.97)	47.81 (44.68 to 50.04)	0.057
Emotion	30.46 (29.89 to 31.01)	43.06 (37.80 to 48.38)	<0.001
Word count	345 (340 to 351)	603 (583.01 to 622.49)	<0.001
Anger	3.00 (3.00 to 3.00)	3.00 (3.00 to 4.00)	0.001
Anticipation	4.00 (4.00 to 4.00)	6.00 (6.00 to 7.00)	<0.001
Disgust	3.00 (2.00 to 3.00)	2.00 (2.00 to 2.00)	<0.001
Fear	6.00 (6.00 to 6.00)	5.00 (4.00 to 5.00)	0.002
Joy	3.00 (3.00 to 3.00)	4.00 (4.00 to 5.00)	<0.001
Sadness	5.00 (5.00 to 5.00)	5.00 (4.00 to 5.00)	0.789
Surprise	1.50 (1.00 to 2.00)	2.00 (1.00 to 2.00)	0.012
Trust	6.00 (6.00 to 6.00)	11.00 (10.00 to 12.00)	<0.001
Positive valence	13.00 (12.00 to 13.00)	22.00 (20.00 to 23.00)	<0.001
Negative valence	9.00 (8.00 to 9.00)	10.00 (9.00 to 11.00)	<0.001

*Mann-Whitney test

Supplementary Figure S1. Total word count for each sentiment category for medical and non-medical PLSs.

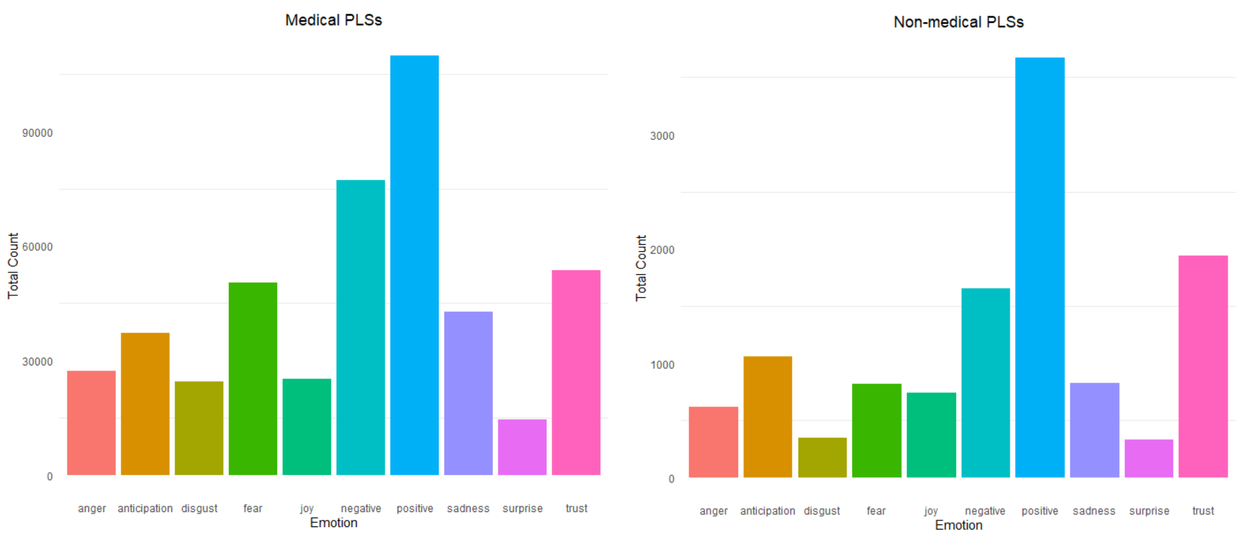

Supplementary Figure S2. Wordcloud representing the most used words from all sentiment categories for medical PLSs.

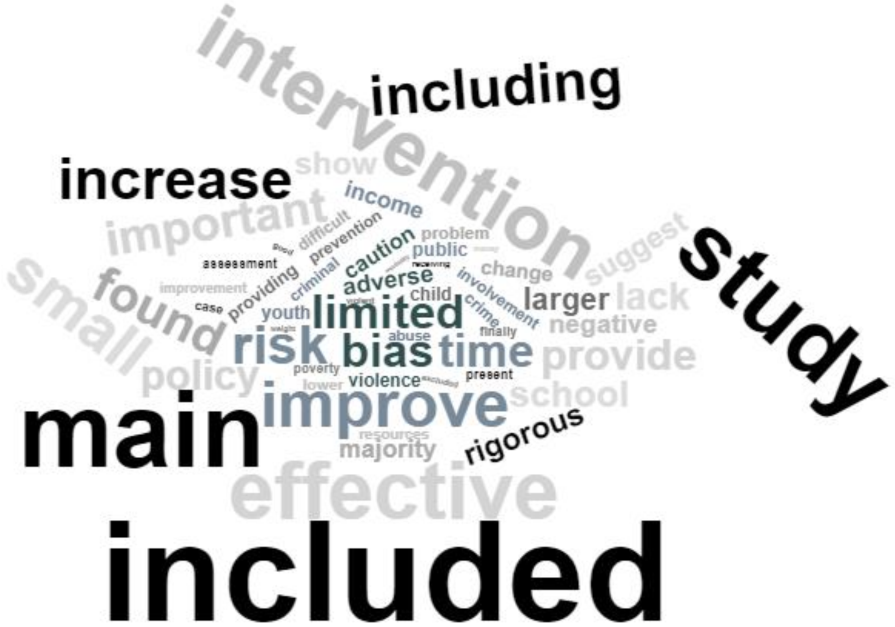

Supplementary Figure S3. Wordcloud representing the most used words from all sentiment categories for non-medical PLSs.

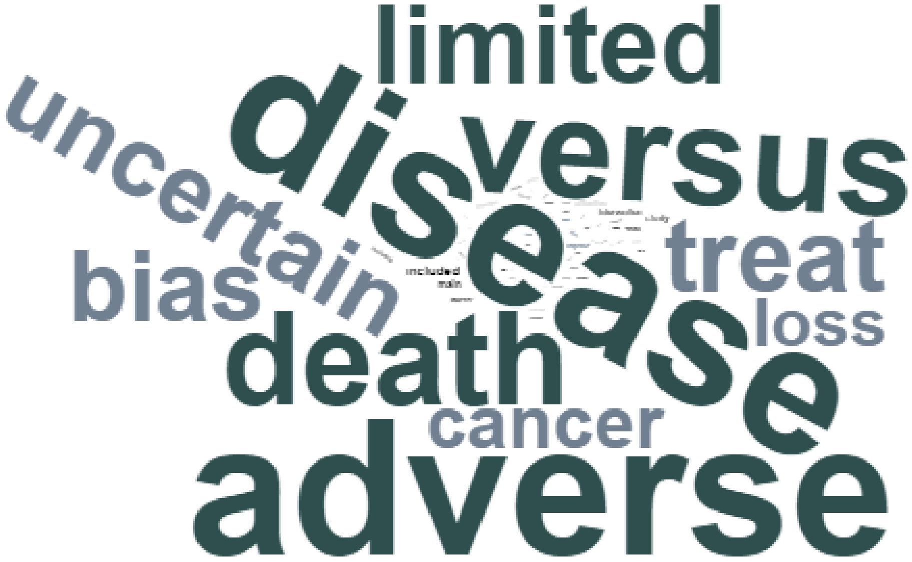

Supplementary Table S19. STROBE Statement—Checklist of items that should be included in reports of ***cross-sectional studies***

	Item No	Recommendation	Page
**Title and abstract**	1	(*a*) Indicate the study's design with a commonly used term in the title or the abstract	1
		(*b*) Provide in the abstract an informative and balanced summary of what was done and what was found	2
**Introduction**			
Background/rationale	2	Explain the scientific background and rationale for the investigation being reported	3-6
Objectives	3	State specific objectives, including any prespecified hypotheses	6
**Methods**			
Study design	4	Present key elements of study design early in the paper	13
Setting	5	Describe the setting, locations, and relevant dates, including periods of recruitment, exposure, follow-up, and data collection	13-14
Participants	6	(*a*) Give the eligibility criteria, and the sources and methods of selection of participants	13
Variables	7	Clearly define all outcomes, exposures, predictors, potential confounders, and effect modifiers. Give diagnostic criteria, if applicable	14-16
Data sources/ measurement	8*	For each variable of interest, give sources of data and details of methods of assessment (measurement). Describe comparability of assessment methods if there is more than one group	14-16
Bias	9	Describe any efforts to address potential sources of bias	11
Study size	10	Explain how the study size was arrived at	NA
Quantitative variables	11	Explain how quantitative variables were handled in the analyses. If applicable, describe which groupings were chosen and why	16-17
Statistical methods	12	(*a*) Describe all statistical methods, including those used to control for confounding	16-17
		(*b*) Describe any methods used to examine subgroups and interactions	NA
		(*c*) Explain how missing data were addressed	NA
		(*d*) If applicable, describe analytical methods taking account of sampling strategy	NA
		(*e*) Describe any sensitivity analyses	NA
**Results**			
Participants	13*	(a) Report numbers of individuals at each stage of study—eg numbers potentially eligible, examined for eligibility, confirmed eligible, included in the study, completing follow-up, and analysed	6-7
		(b) Give reasons for non-participation at each stage	6-7
		(c) Consider use of a flow diagram	7
Descriptive data	14*	(a) Give characteristics of study participants (eg demographic, clinical, social) and information on exposures and potential confounders	NA
		(b) Indicate number of participants with missing data for each variable of interest	NA
Outcome data	15*	Report numbers of outcome events or summary measures	7-8
Main results	16	(*a*) Give unadjusted estimates and, if applicable, confounder-adjusted estimates and their precision (eg, 95% confidence interval). Make clear which confounders were adjusted for and why they were included	7-8 Suppl.
		(*b*) Report category boundaries when continuous variables were categorized	NA

		(*c*) If relevant, consider translating estimates of relative risk into absolute risk for a meaningful time period	NA
Other analyses	17	Report other analyses done—eg analyses of subgroups and interactions, and sensitivity analyses	8
**Discussion**			
Key results	18	Summarise key results with reference to study objectives	8-11
Limitations	19	Discuss limitations of the study, taking into account sources of potential bias or imprecision. Discuss both direction and magnitude of any potential bias	11
Interpretation	20	Give a cautious overall interpretation of results considering objectives, limitations, multiplicity of analyses, results from similar studies, and other relevant evidence	12
Generalisability	21	Discuss the generalisability (external validity) of the study results	12
**Other information**			
Funding	22	Give the source of funding and the role of the funders for the present study and, if applicable, for the original study on which the present article is based	22

Note: An Explanation and Elaboration article discusses each checklist item and gives methodological background and published examples of transparent reporting. The STROBE checklist is best used in conjunction with this article (freely available on the Web sites of PLoS Medicine at http://www.plosmedicine.org/, Annals of Internal Medicine at http://www.annals.org/, and Epidemiology at http://www.epidem.com/). Information on the STROBE Initiative is available at www.strobe-statement.org.
